# On the Origin of Temperature Induced Performance Degradation of Cu-Contacted Mg_2_*X*-Based (*X* = Si, Sn) Thermoelectric Materials

**DOI:** 10.1021/acsami.5c00258

**Published:** 2025-04-29

**Authors:** Radhika Deshpande, Amin Bahrami, Frederic Kreps, Ran He, Pingjun Ying, Kornelius Nielsch, Eckhard Müller, Johannes de Boor

**Affiliations:** † Institute of Materials Research, German Aerospace Centre (DLR), 51147 Cologne, Germany; ‡ Leibniz Institute of Solid State and Materials Science, 01069 Dresden, Germany; § Institute of Materials Science, Technische Universität Dresden, 01062 Dresden, Germany; ∥ Institute of Inorganic and Analytical Chemistry, Justus Liebig University of Giessen, 35392 Giessen, Germany; ⊥ Institute of Technology for Nanostructures (NST) and CENIDE, Faculty of Engineering, University of Duisburg-Essen, 47057 Duisburg, Germany

**Keywords:** thermoelectricity, Mg_2_Si, electrode, contact, degradation, atomic layer deposition, wavelength-dispersive spectroscopy

## Abstract

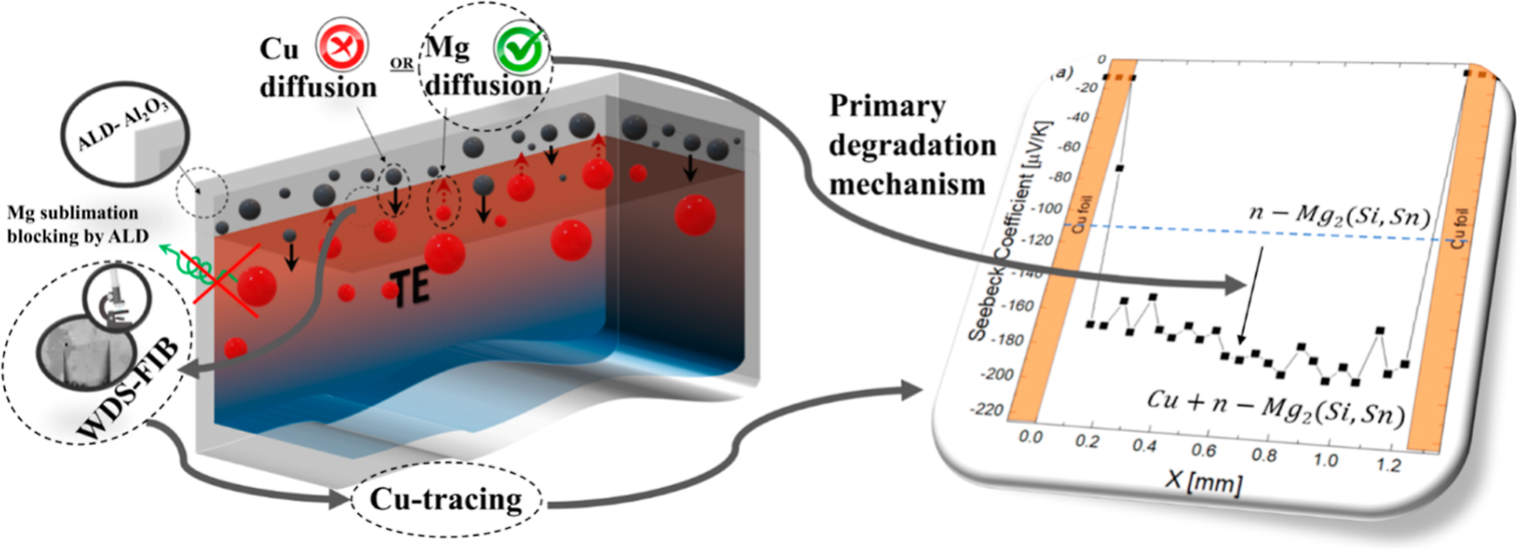

Mg_2_*X* (*X* = Si, Sn)-based solid solutions are promising candidates for mid-to-high temperature waste heat recovery, but developing long-term stable thermoelectric generators (TEG) remains challenging. While Mg_2_*X* demonstrates excellent thermoelectric performance, it is susceptible to Mg loss, causing changes in carrier concentration through the formation of intrinsic defects. More severe degradation has further been observed for samples following contacting with a metal electrode, presenting an additional challenge for TEG technology advancement. We propose atomic layer deposition (ALD) to first enhance the material stability by suppression of Mg sublimation and second to differentiate between the different degradation mechanisms occurring at high operating temperatures. Our primary results from integral electrical conductivity and Seebeck coefficient measurements on coated samples without a Cu electrode at 723 K show enhanced material stability, while spatially resolved interface characterization of samples with Cu electrodes indicate comparable degradation for both coated and uncoated samples. This confirms Mg–Cu interdiffusion as the primary degradation mechanism for contacted samples with a higher relevance than Mg sublimation. Finally, wavelength-dispersive spectroscopy is used to reveal that the observed reduction in the charge carrier concentration during contacting is primarily due to Mg diffusing into the Cu electrode, not by the diffusion of Cu into the TE material as speculated previously, making this study the first to experimentally demonstrate the relevance of Mg loss into the electrode. By combining ALD coating to inhibit Mg loss with microstructural analysis, we present a methodology to distinguish TE material/electrode-related degradation mechanisms, enabling future advancements in Mg_2_(Si, Sn)-based TEGs.

## 1 Introduction

Over the past few decades, there has been substantial growth in the field of thermoelectricity. Thermoelectric generators (TEG) provide a clean energy solution by directly transforming heat into electricity. TEG are compact, durable solid-state devices without any moving components, ensuring noise-free and vibration-free operation, thereby offering reliability over extended periods without requiring maintenance. Capitalizing on these advantages, TEG have found applications in diverse fields such as deep space missions, remote data communication and navigation systems, polar weather stations, and waste heat harnessing endeavors within the aerospace and automotive industries.^1−3^ In 2021, TEG were tested at the Bottle Rock geothermal field in the Geysers region, CA, USA, to use heat sources from geothermal wells, exploring their potential for large-scale application of this technology.^4^ Furthermore, TEG have been implemented in wearable electrocardiographic systems, which are operated by extracting human body heat.^5^

The performance of TEG is determined by the figure of merit (ZT) of the device, which is calculated using eq 1(6)
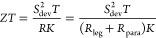
1where *S*_dev_ is the Seebeck coefficient of the device, which is the sum of average values of the Seebeck coefficients of n- and p-type thermoelectric legs employed in the device, *R* is the total resistance which contains the contributions from the TE legs *R*_leg_ and additional resistances (e.g., from contacts), summarized here as *R*_para_. *K* contains the total thermal conductance of the legs *K*_leg_ and might also consider further parasitic heat bypasses, and *T* is the absolute temperature. The device *ZT* can be approximated by the figure of merit *zT* of the thermoelectric (TE) materials, given by eq 2, only when contact resistance and further losses in the device are negligibly small and the p- and n-type materials exhibit similar thermoelectric properties. This approximation represents an upper ideal limit.
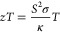
2where *S*, σ, *T*, and κ are the Seebeck coefficient, the electrical conductivity, the absolute temperature, and the thermal conductivity, respectively.

A wide range of TE materials and their integration into operational TEG has been studied such as Bi_2_Te_3_ or PbTe. Long-term stability is often cited as a primary advantage of TE systems, but TE materials can degrade, primarily through thermal decomposition or sublimation. This is particularly relevant for recently developed high-performance materials, which, due to their intrinsic chemical and structural complexity, exhibit enhanced performance but face compromised thermal stability simultaneously. Besides easily observable changes such as surface oxidation, changes in the charge carrier concentration of the material resulting from interactions with the environment present a significant challenge, as these are not readily evident through conventional microstructural characterization techniques. At the device level, additional complexities arise with the formation of extensive interdiffusion zones (IZ) that can consume the TE material by chemical reaction or degrade it. IZ refer to the region around the interface between a semiconductor and a metal electrode, where diffusion and reaction phenomena take place. Often, it is confined to a few μm in thickness, but for example, in the case of Cu and Mg_2_(Si and Sn), the IZ can exceed 100 μm^7^. On the other hand, diffusion processes may not lead to visible chemical reactions but can still induce changes in defect concentration well beyond the range of the IZ as has been observed for various material classes.^8,9^ In summary, the following three mechanisms are potentially relevant for functionalized TE legs or modules but not always easily distinguishable:

(i) Diffusion of elements toward the surface and subsequent sublimation or reaction such as oxidation,
(ii) Diffusion of atoms from the electrode into the TE material, resulting in the formation of defects there,
(iii) Diffusion of elements from the TE material toward the electrode leading to the formation of (further) point defects inside the TE material.

Sublimation and change of TE properties without decomposition was observed for various materials such as Mg loss for Mg_3_Sb_2_^10^ and Mg_2_(Si, Sn),^7,8,11^ Sb sublimation in Skutterudites^12^ and Te loss from BiTe,^13,14^ usually leading to performance degradation in the long run. However, for functionalized legs as required for device fabrication, different and often faster degradation was observed without identifying the underlying mechanism.^11,15,16^ This prevents the effective development of counter measures and, consequently, there is a keen interest in differentiating the different mechanisms and developing experimental procedures to achieve it.

**Figure 1:**
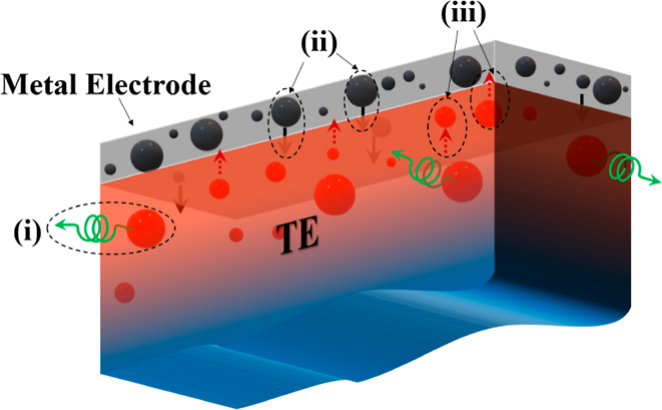
Illustration of point-defect-related plausible mechanisms leading to a degradation of functionalized TE legs: (i) component loss, e.g., by diffusion toward the surface, (ii) diffusion of electrode atoms into the TE material, and (iii) diffusion of TE material atoms into the electrode metal.

In this paper, we study subtle intricacies associated with Cu contacts interfacing with n-type Mg_2_(Si, Sn). Mg_2_*X* (*X* = Si, Sn)-based solid solutions are an attractive class of materials for mid-to-high temperature waste heat recovery applications.^17−20^ These materials possess a high thermoelectric figure of merit (n-type: *zT* ≈ 1.4 at 723 K^17^ and p-type: *zT* ≈ 0.55 at 623 K^18^) and are non-toxic, lightweight, abundant, and affordable. There have been a few reports on the use of these materials for the fabrication of thermoelectric modules,^21−23^ and three fully Mg_2_(Si, Sn)-based TEG have been reported previously. In 2016, Gao reported to have achieved a maximum power output of 0.12 W with a corresponding power density of 0.47 W/cm^2^ for *T*_h_ = 713 K and *T*_c_ = 383 K.^24^ In 2021, Goyal and Dasgupta *et al.* had also reported a power density of 0.52 W/cm^2^ (with respect to the area of the TE legs) and predicted a maximum efficiency of 5%.^25^ Later in 2023, Camut *et al.* reported a power density of 0.9 W/cm^2^ (TE area) and a maximum efficiency of 3.6% for a full Mg_2_(Si, Sn) module.^15^ Most recently, Deshpande *et al.*(26) reported a maximum power of ∼0.79 W/cm^2^ (TE area) for a silicide-based TEG, wherein (doped) binary Mg_2_Si was used as a n-type counterpart to p-type Mg_2_(Si, Sn). This substitution of the popular n-type solid solution was proposed due to the observed degradation caused by Mg loss and contact–electrode interdiffusion. With respect to contacting, the case of Cu as an electrode is particularly intriguing, as it is the standard material for the bridge, which connects the legs of the TE module because of its high electrical conductivity. It is also known for providing good adhesion due to its coefficient of thermal expansion (CTE) closely matching that of the given TE material (Cu: 17 × 10^–6^ K^–1^^27^ and Mg_2_Si_0.3_Sn_0.7_: 17.5 × 10^–6^ K^–1^^28^). Prior research by Ayachi *et al.*(7) reported low values of electrical contact resistance for both n- and p-type silicides (<10 μΩ·cm^2^) with the occurrence of wide, highly conductive diffusion regions. However, after annealing, the contact resistance for n-type silicide in contact with Cu increased to ∼100 μΩ·cm^2^, whereas for p-type, it remained consistently low (<10 μΩ·cm^2^). They also observed the formation of a thick IZ and a change in the Seebeck coefficient of the TE material well beyond the IZ, indicating a change in charged defect concentration. This highlights that while Cu serves as a reliable contact for p-type Mg_2_(Si, Sn), diffusion-related degradation poses challenges for n-type. While the formation of Cu-related defects in Mg_2_*X* has been hypothesized based on calculated defect formation energies by Ayachi *et al.*,^29^ the experimental verification of this is missing and, if confirmed, it furthermore remains unclear whether this is the only reason for the observed material change. In accordance with the earlier described mechanisms (Figure 1), further possibilities include Mg diffusion to the surface and subsequent sublimation as well as Mg diffusion into the electrode, both modifying the TE properties by changing the density of charged, Mg-related defects. Recent studies have revealed high diffusivity of Mg,^30,31^ suggesting that the stability of Mg-rich Mg-based materials may be compromised even at room temperature.

This work therefore involves subjecting Cu-contacted samples to annealing conditions at 723 K for the duration of 1 week, aligning with the operating temperature profile anticipated for the final Mg_2_*X*-based thermoelectric device. The main focus of this investigation is the identification and discrimination of degradation mechanisms, as visualized in Figure 1. The scientific approach involves application of an alumina coating via atomic layer deposition (ALD), followed by high-temperature annealing under an inert atmosphere. This method is effective for differentiation of various degradation mechanisms. Integral TE measurements conducted before and after annealing of the coated, non-contacted bulk TE sample provide the evidence that Mg loss via sublimation can be effectively suppressed by ALD coating. Additionally, local Seebeck coefficient measurements on Cu-contacted samples reveal alterations in the carrier concentration, indicating a change in defect concentrations in the coated and annealed samples. Using wavelength-dispersive spectroscopy (WDS), we find no indications for Cu diffusion into the TE material. These findings collectively suggest that the primary degradation mechanism is the diffusion of Mg into the electrode, rather than Cu diffusion into the TE or Mg loss through sublimation, providing the base for effective countermeasures.

## 2 Experimental Section

The synthesis of n-type TE powder was performed by using the melting route introduced in refs (7, 17, and 32) with the nominal stoichiometry Mg_2.06_Si_0.3_Sn_0.665_Bi_0.035_. Excess Mg (3 at.% with respect to nominal Mg content) was added to compensate for potential Mg loss during the preparation steps in order to obtain nearly phase-pure materials. The powders were sintered in a direct current sintering press (DSP 510 SE, Dr. Fritsch GmbH) with a heating rate of 1 K/s, and the pressing conditions (*T* = 973 K for *t* = 1200 s, *P* = 66 MPa) were optimized based on previous research to achieve optimal TE properties. Two batches (b1 and b2) with slightly different carrier concentrations were employed for this study. Density measurements were performed using Archimedes’ method, yielding a relative density of >95% for all samples. The temperature-dependent electronic transport properties were measured using a four-probe technique in an in-house-developed facility.^33,34^ Electrical contact to the coated samples was enabled by the usage of pressure contacts and local scratching of the sample while mounting into the holder.

The n-type TE material was joined to Cu by stacking Cu foils (99.9%, Goodfellow) on either side of the TE pellet and exposing the stack to 873 K and 29 MPa for 10 min in the DSP facility. The number of foils stacked on each side was chosen to ensure an electrode thickness of at least 100–200 μm in the final sample. Depending on availability, either a single 250 μm foil or a stack of 3 foils of 50 μm each side were used to achieve this. The final thickness varies due to the reaction with Mg_2_*X* and the polishing required to remove graphite from the sample after the joining step. The optimal compaction temperature for Mg_2_Si_1–*x*_Sn_*x*_ depends on the solid solution composition *x*^18^, and the suitable processing temperature window to join the metal electrode to the TE material is also specific to both components. The Potential and Seebeck scanning microprobe (PSM) was employed to scan the surfaces of the contacted samples to map the values of the Seebeck coefficient at room temperature before and after annealing.^35^ Note that thermal conductivity measurements are not possible on contacted samples due to the sample dimensions; therefore, these are not included here.

Al_2_O_3_ thin films were grown by ALD in a Savannah 200 reactor from Veeco using Trimethylaluminum (TMA, 98%, Stem Chemicals) as the aluminum precursor and ultrapure water as an oxygen source. The TMA and water canisters were left at room temperature. The reaction chamber temperature (*T*_ALD_) was set to 423 K. All the thermoelectric pieces were held from the contact sides or the smallest surface side faces (∼1.5 mm^2^) in case of the non-contacted samples, exposing all other sides to the TMA and water. The ALD cycle consisted, for both TMA and H_2_O, of sequential pulses and long purge steps. The pulse and purge durations were 0.2:20 s for both TMA and water. As the reference sample, a Si(100) wafer was cut into 10 × 10 mm^2^ squares and placed in different parts of the ALD chamber for thickness measurement using a spectroscopic ellipsometer (SENpro, SENTECH). Having the growth per cycle value obtained from preliminary tests (∼0.11 Å/cycle), 3000 ALD cycles were done to achieve *a* ∼ 330 nm Al_2_O_3_ layer.

For annealing, the ALD-coated samples, both non-contacted and Cu-contacted, were positioned in the holder of an in-house built vertical furnace. It employs the principle of radiation heating and features an elongated tube furnace to ensure uniform temperature distribution around the sample. Subsequently, they underwent a heating process to 723 K, with a ramp rate of 5 K per minute, and were maintained at this temperature for a duration of 170 h within an Ar atmosphere. Following the annealing process, the samples underwent passive cooling down before being subjected to characterization.

X-ray diffraction (XRD) was conducted on sintered, ALD-coated, and annealed pellets using a Siemens D5000 Bragg–Brentano diffractometer. The system employed Cu Kα radiation (1.5406 Å) over a 2θ range of 20° to 80° and a grazing incidence of 0.2°, with a step size of 0.02° at 20 s per step. Subsequently, these samples were further examined using a scanning electron microscope (Zeiss Ultra 55c) equipped with an energy-dispersive X-ray (EDS) Spectrometer, employing a 15 kV acceleration voltage. However, EDS has limitations in detecting trace elements below 1.0 wt %. For such analyses, wavelength-dispersive X-ray spectrometry (WDS) is preferred due to its higher energy resolution, increased accuracy, and detection limits below 0.1 wt %. WDS was employed to scan the surfaces of the cross-sectioned contact face at and near the interface to quantify the atomic fraction of Cu. To achieve high performance with WDS, samples must be homogeneous, smooth, conductive, non-contaminated, and positioned at a 90° angle to the electron beam. The WDS system, integrated with a dual-beam microscope and a gallium ion beam, allows in situ cleaning of the sample surface. The high vacuum environment prevents contamination and oxidation post cleaning. This procedure ensures the highest accuracy in WDS analysis, enabling the reliable detection of trace elements. The measurement was conducted using a Thermo Fisher Scientific MagnaRay Wavelength Dispersive X-ray Spectrometer equipped with a Lithium Fluoride crystal (LiF) crystal, operating at 15 keV and a 1.4 nA aperture. This spectrometer is integrated into the FEI Helios NanoLab 600i Dualbeam Electron Microscope (Focused Ion Beam). The non-contacted samples were then subjected to an assessment of their bulk electronic transport properties directly with the coating in place. The thermocouple tips were gently pressed locally into the ALD coating layer to establish a direct contact with the TE material.

## 3 Results

Table 1 presents the series of samples, used in this work along with their nominal compositions, processing conditions and RT Seebeck coefficients. The labeling system uses the following format: sample number—powder batch number (b1/b2)—A for ALD—a for annealing—c for contacting. The ’b1/b2′ denotes different powder batches, with starting Seebeck coefficients of −110 μV/K for b1 and −91 μV/K for b2.

**Table 1:** Nominal Sample Compositions along with Sample Processing^a^

sample name	composition	ALD	annealing	*S* (μV/K)
0-b1	Mg_2.06_Si_0.3_Sn_0.665_Bi_0.035_	X	X	–110
1-b1-A	Mg_2.06_Si_0.3_Sn_0.665_Bi_0.035_	√	X	–110
2-b1-A-a	Mg_2.06_Si_0.3_Sn_0.665_Bi_0.035_	√	√	–110
3-b1-c	Mg_2.06_Si_0.3_Sn_0.665_Bi_0.035_ + Cu on both sides	X	X	–178
3-b1-c-A-a	Mg_2.06_Si_0.3_Sn_0.665_Bi_0.035_ + Cu on both sides	√	√	–191
4-b2	Mg_2.06_Si_0.3_Sn_0.665_Bi_0.035_	X	X	–91
4-b2-c	Mg_2.06_Si_0.3_Sn_0.665_Bi_0.035_ + Cu on both sides	X	X	–140
5-b2-c	Mg_2.06_Si_0.3_Sn_0.665_Bi_0.035_ + Cu on one side	X	X	–110/–95

a The fifth column lists the Seebeck coefficient at RT after the specific procedures applied to each sample. For 5-b2-c, the values for both contacted and non-contacted interfaces are given.

**Figure 2:**
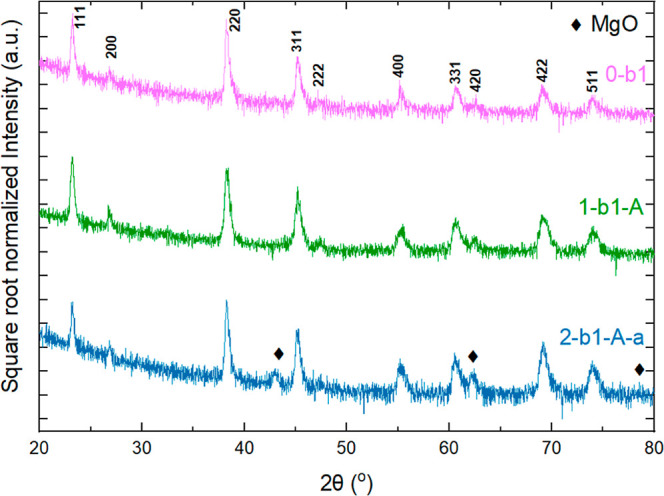
Grazing incidence XRD scans of the sintered reference (0-b1), ALD-coated (1-b1-A), an ALD coated + annealed (2-b1-A-a) samples. The intensity is plotted as a square root of the normalized intensity.

Figure 2 presents grazing incidence XRD measurements conducted on bulk surfaces of n-Mg_2_(Si, Sn) in three states and labeled as follows: sintered (0-b1), coated by ALD (1-b1-A), and annealed (with coating 2-b1-A-a). The 0-b1 sample serves as a reference to identify any alterations in microstructure resulting from coating or annealing at 723 K for 1 week. The (*hkl*) indices have been labeled for all visible peaks in the 0-b1 sample. In addition to GIXRD (Figure 2), a bulk XRD measurement was conducted on the same 0-b1 sample and is reported in the SI Figure S1. It is important to note that GIXRD and XRD were performed using Cu (λ = 1.5406 Å) and Co (λ = 1.7902 Å) sources, respectively, to achieve better resolution of the peaks and due to instrument availability. All peaks observed for sample 0-b1 can be indexed to Mg_2_*X* with the cubic *Fm*3̅*m* space group. From both Figure 2 and Figure S1, it is evident that the 0-b1 sample is free from impurities such as Si or MgO (for peak positions, see JCPDS 4-829^36−38^). There are no additional peaks in sample 0-b1, however, MgO reflections emerge for the 2-b1-A-a sample, indicating the formation of MgO on the surface as a consequence of the annealing process. Due to the grazing incidence of 0.2°, the measurement signal stems only from the sample surface (∼600 nm penetration depth), making surface changes much more visible than standard XRD.

**Figure 3:**
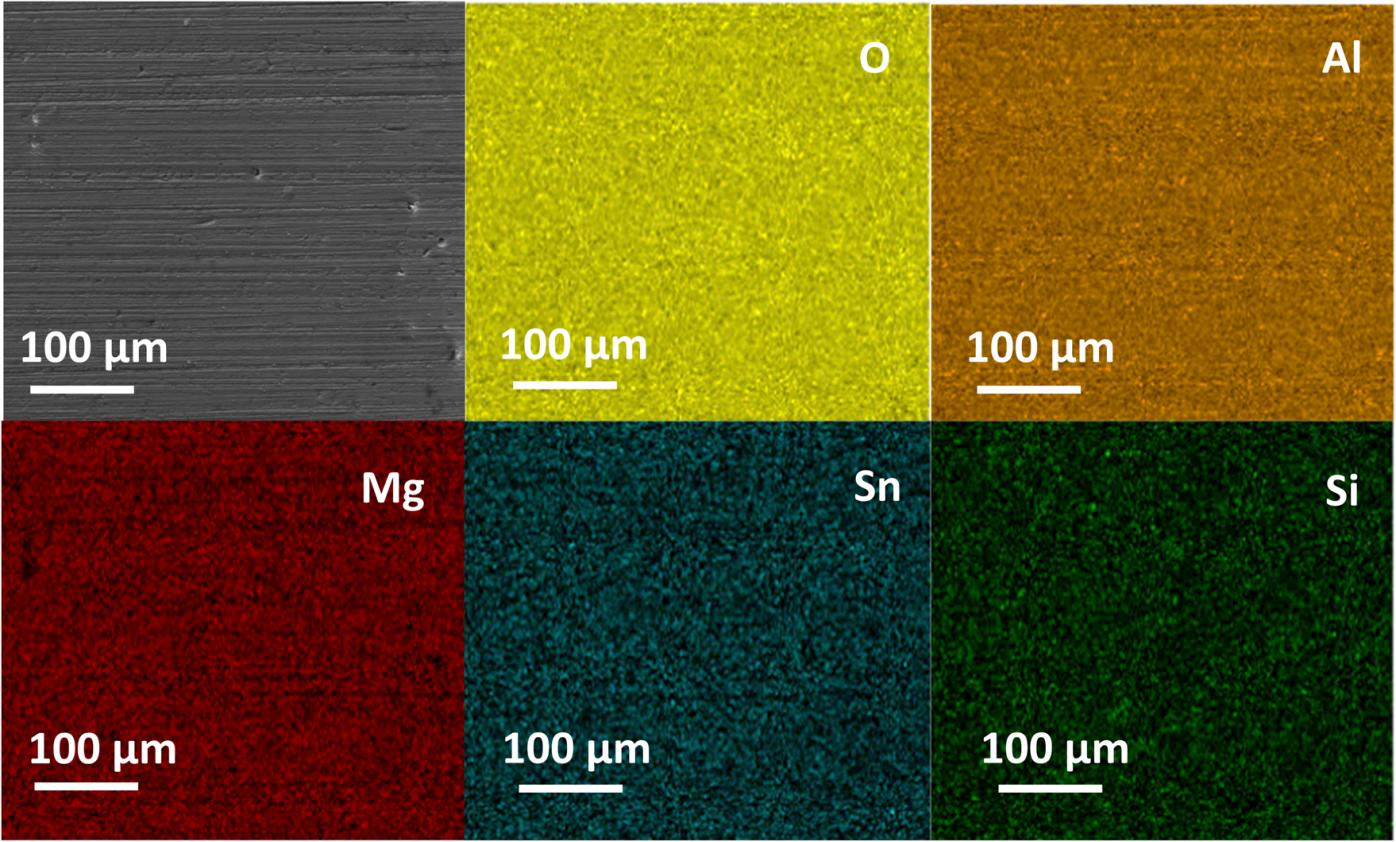
Low magnification BSE and EDX elemental map for 1-b1-A proving uniform coating of the sample by Al_2_O_3_.

An SEM image of the 1-b1-A sample is shown in Figure 3. A low magnification EDX elemental map was generated to evaluate the uniformity of the coating, revealing that Al and O are evenly distributed across the targeted area. The map also reveals a dense and uniform coating with no visible cracks. We have provided the mass % of an EDS point analysis of Figure 3 in the Supporting Information (Table S1) and note that Si signals beneath the Al_2_O_3_ coating are weak. However, Sn and Mg signals are strong enough to demonstrate the sample’s homogeneity. To confirm the continuity of the coating layer, we first examined all ALD-coated samples under a light microscope. Samples with uniform coating coverage were selected for further SEM-EDS elemental analysis. The corresponding light microscope image is included in Figure S2a. As shown in Figure S2b,c a contrast in color is observed at the interface where the coating has chipped off, further confirming the uniform coating of the selected samples (Figure 3) for this research.

**Figure 4:**
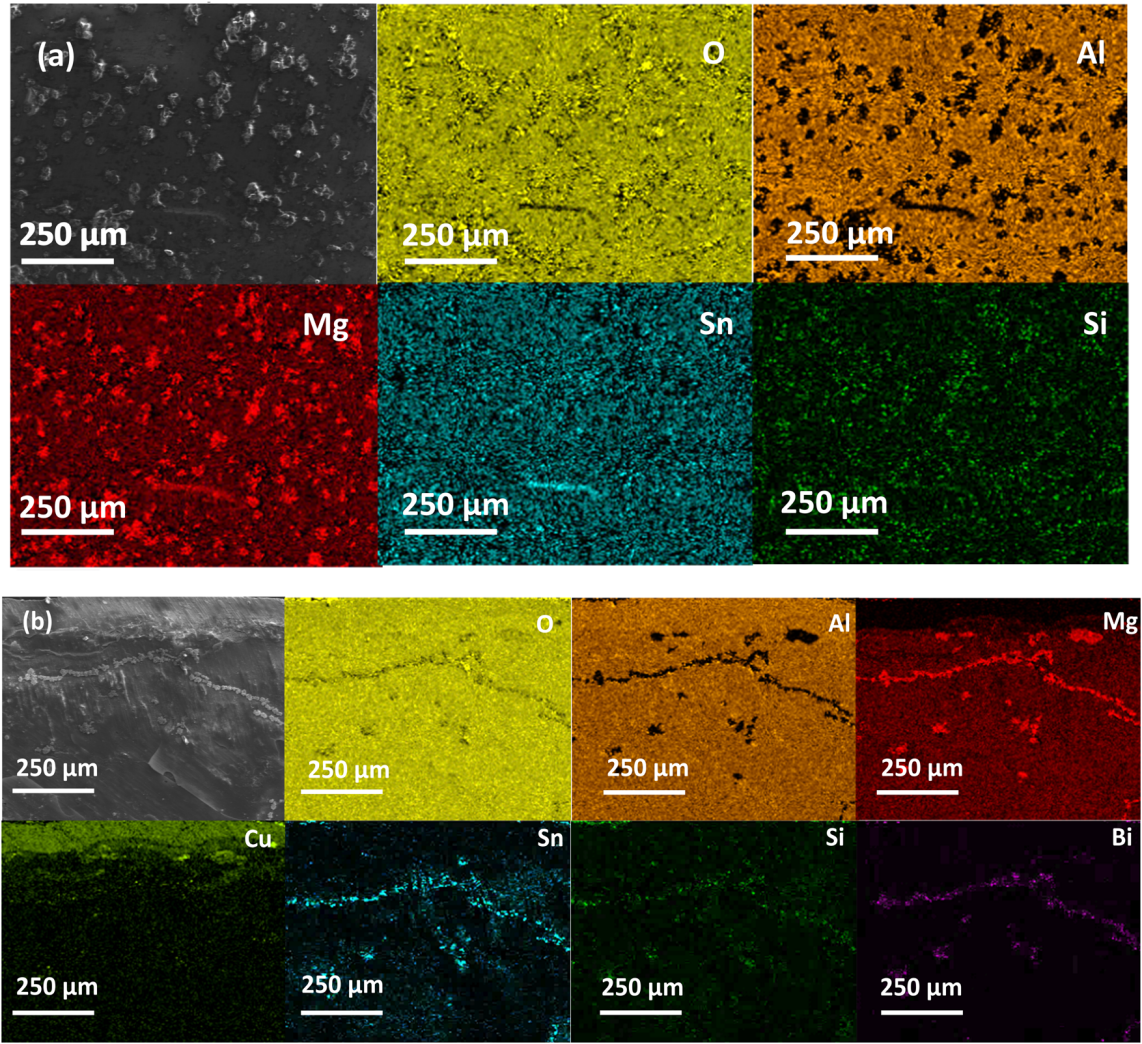
Low magnification elemental map for (a) 2-b1-A-a; (b) 3-b1-c-A-a, showing the cross-sectional face at the interface region between Cu contact and TE material. While most of the coating is still intact, inhomogeneities are clearly visible for both samples, indicating a partial degradation of the Al_2_O_3_ coating.

Figure 4a,b depicts the elemental maps for the samples 2-b1-A-a and 3-b1-c-A-a, i.e., after coating and annealing or, additionally, previous contacting, respectively. For 2-b1-A-a, the presence of MgO islands is evident, and upon closer examination, it becomes apparent that these islands form wherever the coating is breached. Similarly, the 3-b1-c-A-a sample, as illustrated in Figure 4b, shows MgO in locations where the coating is compromised. This phenomenon may arise from the coefficient of thermal expansion (CTE) mismatch between Al_2_O_3_ (8 × 10^–6^ K^–1^ at 450 °C and ∼5 × 10^–6^ K^–1^ at RT^39^) and the TE material (16.5 × 10^–6^ K^–1^^28^) in the specified temperature range. Notably, the topology of the individual MgO islands appears consistent in both samples, however, in the non-contacted sample those are positioned randomly, while in the contacted sample they also form interconnected lines. We cannot definitively conclude at this point whether the CTE mismatch between Al_2_O_3_ and TE material leads to cracking and subsequent localized Mg sublimation followed by MgO formation, or if a potential reaction between sublimating Mg and the Al_2_O_3_ coating to form MgO (CTE ∼ 10.4 × 10^–6^ K^–1^ at RT and 14 × 10^–6^ K^–1^ at 723 K^40^) causes a subsequent cracking of the coating layer. Upon undergoing identical surface treatments, both contacted and non-contacted samples exhibit differences in the quality of the coating after annealing. Specifically, the coating on the non-contacted sample appears more porous than on the contacted sample.

Figure 4a,b also reveals that at the cracked coating sites, there are traces of Sn, Si, and Bi present. This is in partial agreement with the XRD results where reflections of Si (but not of Sn) are observed.

**Figure 5:**
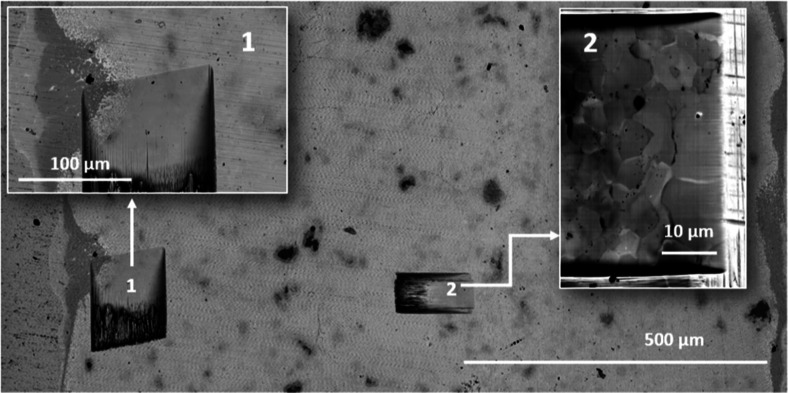
SEM image of FIB-milled areas with high magnification details: (1) near Cu-TE interface and (2) deep within the TE material. WDS measurements were performed in both milled areas.

Figure 5 presents a low-magnification overview of the 3-b1-c sample, highlighting two FIB-milled regions of the sample, which are shown in detail in high-magnification insets: (1) near the Cu-TE interface and (2) deep inside the TE material. The TE material is ∼1.2 mm thick with Cu contacted on both sides. From zoom-in 1, it is also evident that the IZ region contains at least two different layers and a eutectic structure extending toward the TE with a total width locally exceeding 130 μm, indicating significant diffusion of the participating elements and comparable to earlier reports by Ayachi *et al.*(7) Zoom-in 2 shows the sample about 500 μm from the margins. As the sample is freshly ion-milled and the image is captured at high magnification, it reveals the expected grainy structure which was not captured in zoom-in 1, likely due to the vicinity of the IZ, resulting in lower contrast within the TE and fewer observable features.

**Figure 6:**
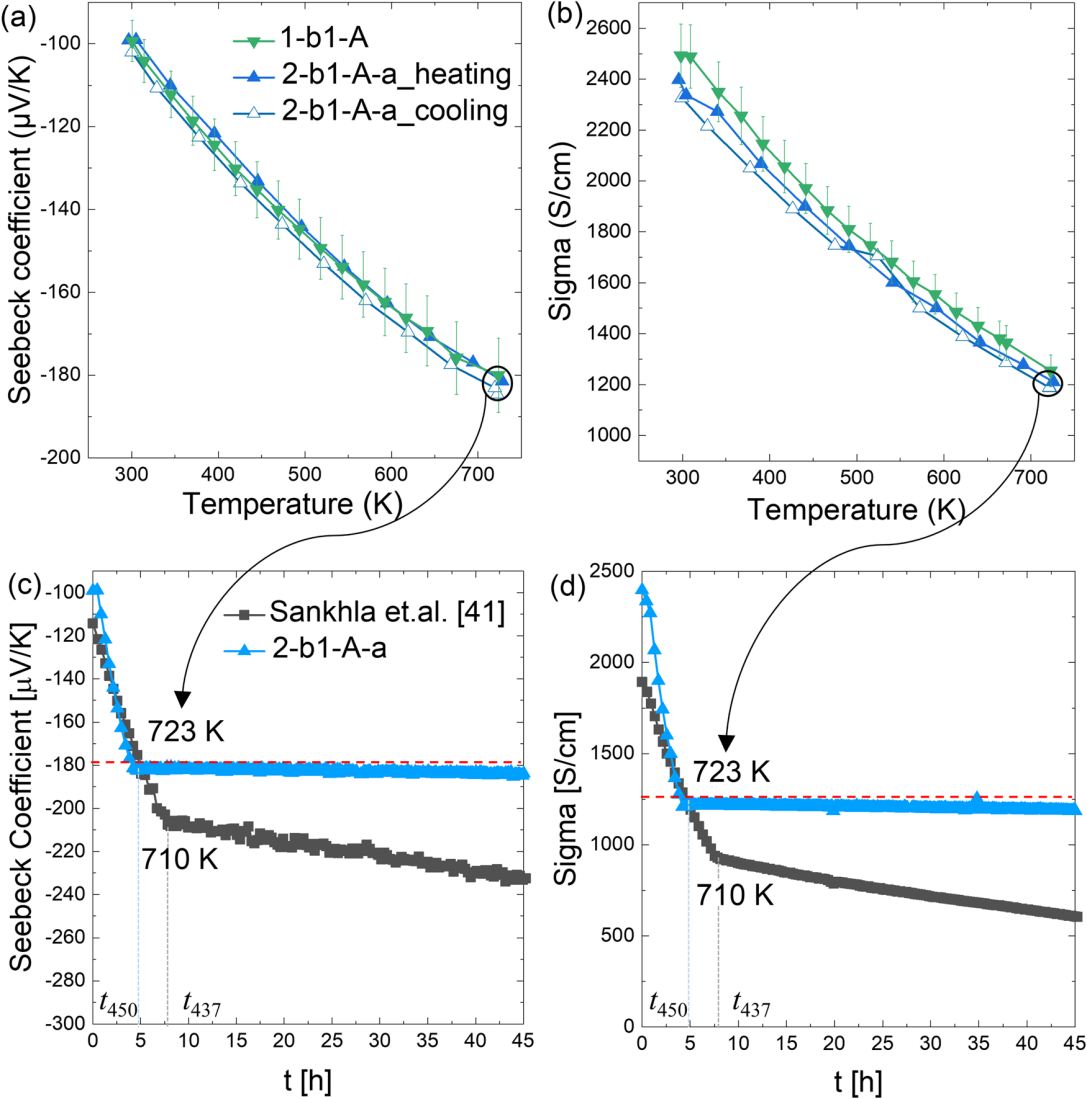
(a,b) Temperature- and (c,d) time-dependent TE data for 1-b1-A and 2-b1-A-a. For sample 2-b1-A-a, a holding time of 45 h was applied at 723 K within the measurement facility during the heating and cooling cycle. This holding time data (Initial heating times for *t*_450_ and *t*_437_, showing different ramp-up rates for each.) is compared in (c,d) with the heat treatment of an unprotected sample at 710 K under similar conditions, demonstrating the enhanced stability of coated samples and the effective inhibition of Mg loss.

Figure 6a,b presents temperature-dependent integral TE properties of n-type samples after coating (but before annealing) (1-b1-A) and for a coated sample after annealing for 1 week (2-b1-A-a): heating and cooling with a holding time of ∼45 h inside the facility in between. Both heating curves are very similar, indicating nearly no sample degradation during the annealing. Additionally, Figure 6c,d presents data recorded during the holding time at 723 K as well as the initial heating up period. For comparison, we also include annealing data at 710 K from Sankhla *et al.*(41) for an n-type sample with a similar composition but without coating. For our sample 2-b1-A-a, the holding measurement starts after the fifth hour, whereas in Sankhla *et al.*’s study, it begins around the eighth hour. This initial change reflects the temperature-dependent characteristic from Figure 6a,b, now represented as a function of time in Figure 6c,d, instead of temperature as shown in (a, b). It can be seen that both the Seebeck coefficient and the electrical conductivity of the uncoated sample change, while for the coated sample the TE properties remain almost unchanged, indicating a strongly improved stability of the material system. As visualized by the red reference line, the changes in both the Seebeck coefficient and electrical conductivity reduced by a factor of 10 for the coated sample. Furthermore, it can be seen that the annealed sample has reverted to the initial room temperature values of the TE properties both after annealing and the subsequent measurement and holding. For comparison, the unprotected sample from Sankhla *et al.* showed a change in *S* from −116 μV/K to −228 μV/K, corresponding to Δ*n* ∼ 2.16 × 10^20^ cm^–3^^41^ at RT after annealing at 710 K for a total of ∼276 h, i.e., under somewhat similar conditions. This underlines the improved stability attained by the coated samples, signifying an effective inhibition of Mg loss.

**Figure 7:**
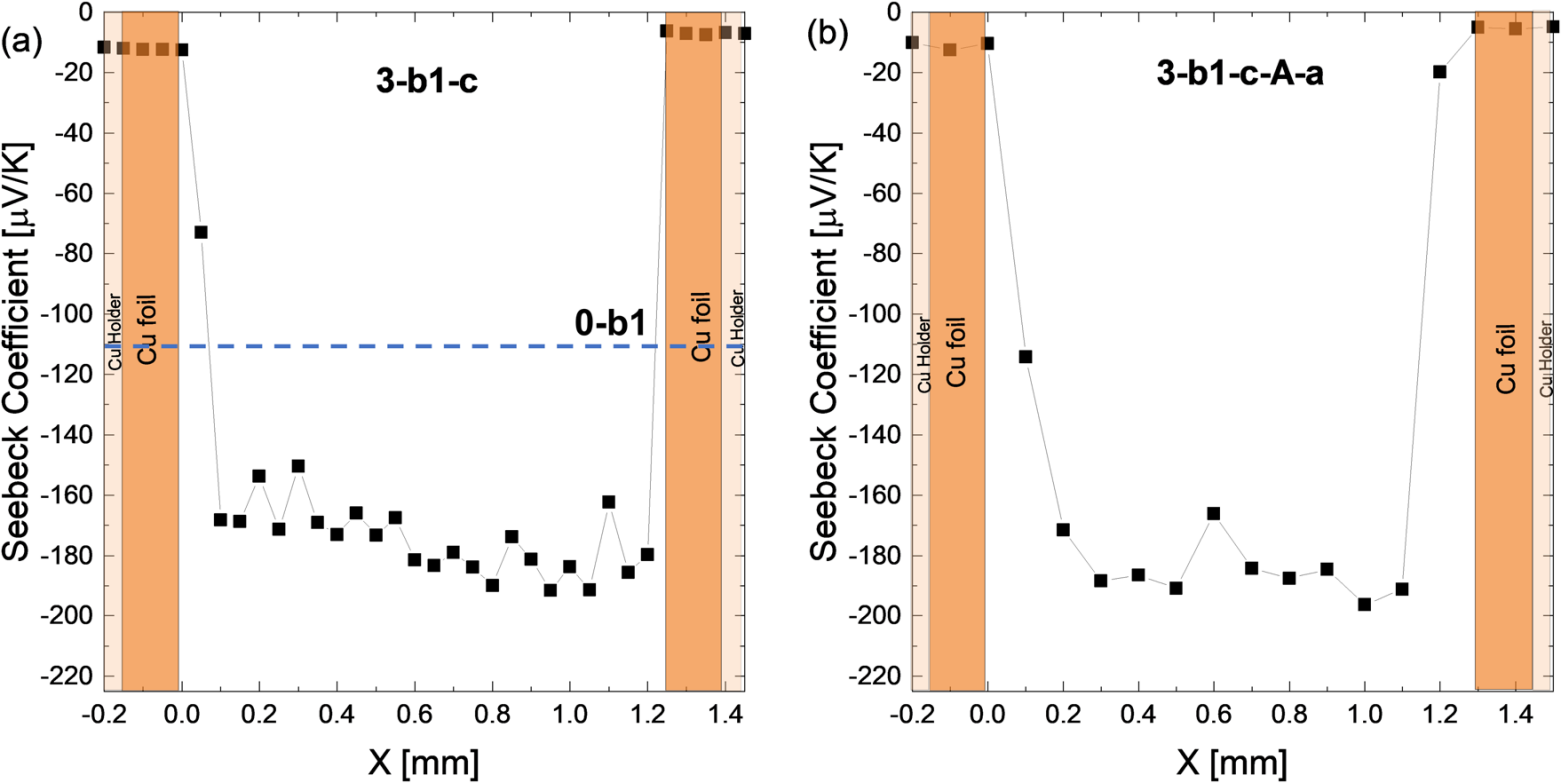
Representative Seebeck coefficient line scans for (a) 3-b1-c and (b) 3-b1-c-A-a; the dashed line corresponds to the bulk value of 0-b1, the reference sample. Sample 3-b1-c-A-a exhibits slightly lower Seebeck values compared to sample 3-b1-c, despite the protective coating during annealing, plausibly suggesting that further Mg–Cu interdiffusion plays a role. “Cu holder” indicates the copper-made brackets of the sample holder of the PSM employed for positioning and electrical contact during measurement.

Figure 7 presents exemplary Seebeck coefficient line scans obtained at room temperature for both 3-b1-c and the coated 3-b1-c-A-a samples, where the latter is the coated and annealed version of the former; this comparison thus allows to identify the effect of joining with Cu and potential further effects of subsequent annealing for a coated sample. Both samples are from batch 1 and the reference sample 0-b1 exhibits a Seebeck coefficient value of approximately −110 μV/K at room temperature. The carrier concentration *n* is estimated using the measured RT Seebeck coefficient and the Pisarenko plot calculated with a single parabolic band model (SPB) for a constant effective mass *m*_D_* = 2.1 *m*_0_,^11,42^ resulting in *n* = 2.2 × 10^20^ cm^–3^. After Cu contacting, a substantially higher (absolute) value of −178 μV/K is found, corresponding to *n* = 0.8 × 10^20^ cm^–3^ indicating a noticeable impact from Cu contacting. With respect to the initially introduced three potential material change mechanisms (Mg loss to the sample surface by sublimation or oxidation, Mg diffusion into Cu, Cu diffusion into TE material), a dominant effect of Cu–Mg interdiffusion is more likely than Mg loss to the surface, given that the sintering process (973 K) prior to the contacting step (873 K) occurred at a higher temperature and both process durations are similar. Furthermore, in a previous study, Sankhla *et al.* demonstrated that only at sintering temperatures beyond 973 K, small changes in carrier concentration (by 6 × 10^18^ cm^–3^) can be observed for the same device as employed here, making Mg loss by sublimation unlikely to be the dominant effect here.^41^

The sample 3-b1-c-A-a exhibits a slightly higher average absolute Seebeck coefficient of *S* ∼ −191 μV/K, compared to the corresponding, non-annealed sample 3-b1-c. The loss of charge carriers Δ*n* due to contacting (1.4 × 10^20^ cm^–3^) is 10-fold higher than the loss after annealing (0.13 × 10^20^ cm^–3^).

The Seebeck coefficient and corresponding charge carrier concentration data show that the contacted sample experiences significant carrier concentration loss which proceeds slowly under annealing (despite the usage of protective coating during annealing) to reach an even lower carrier concentration value. The results indicate that Mg loss by sublimation is not the main cause for this, but rather Mg or Cu diffusion, which leads to a fast change during contacting (873 K, 10 min) and a subsequent slow change during annealing. With the ALD coating preventing Mg loss through the sample surface effectively, the change observed during annealing is plausibly due to further Mg or Cu interdiffusion.

**Figure 8:**
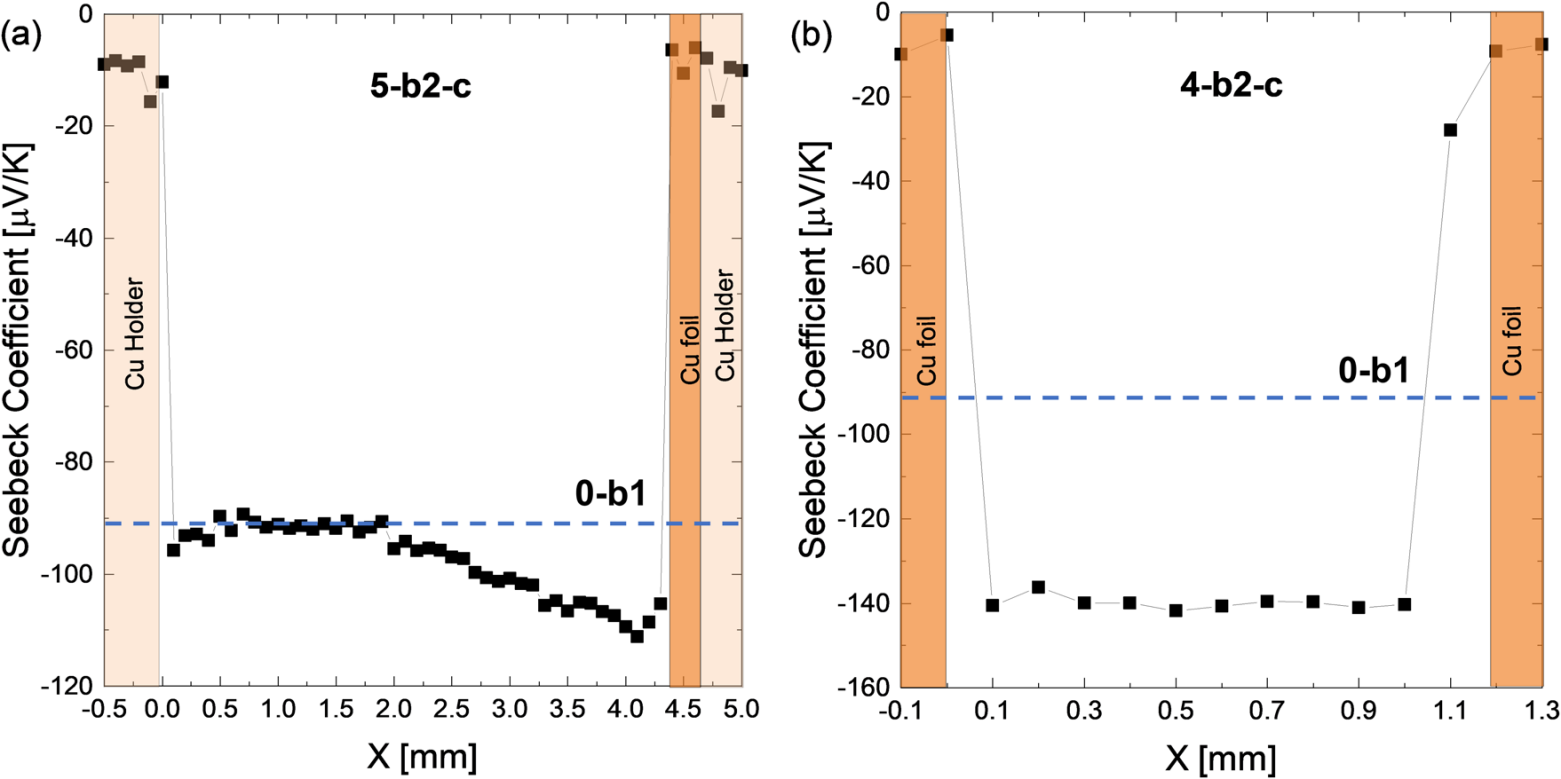
Representative Seebeck profiles for (a) thick one-side contacted sample 5-b2-c; (b) thin two-side contacted sample 4-b2-c. For the thick sample a gradient is clearly visible, for the thin not; presumably because the change in the sample due to the Cu electrode is already saturated. “Cu holder” indicates the copper-made brackets of the sample holder of the PSM employed for positioning and electrical contact during measurement.

A typical indication for electrode-induced defect formation in the TE material by elemental diffusion is a gradient in the Seebeck coefficient close to the interface as observed by Ayachi *et al.* and others.^8,11,29^ It reveals changes in carrier concentration, typically showing a loss of carriers accompanied by an increase in the absolute Seebeck coefficient value. On the other hand, if Mg diffuses out of the TE, it should also result in a reduction in charge carrier concentration as Mg loss corresponds to the annihilation of Mg interstitials (donor defects) or the creation of Mg vacancies (acceptor defects),^30,43^ which should be reflected in a similar gradient in Seebeck coefficient line scans. Such a gradient is not observed in Figure 7, potentially because the sample is relatively thin and the interdiffusion processes due to contacting and annealing might have already led to a (new) equilibrium state after contacting. To clarify, a thick TE sample (Batch b2, with *S* ∼ −91 μV/K before contacting, corresponding to *n* = 3.1 × 10^20^ cm^–3^) was contacted on a single side and clearly displays a gradient on the Cu-contacted side as visible in Figure 8a, confirming the earlier suspicion that Cu or Mg interdiffusion is the main degradation mechanism. We observe a diffusion length of around 2 mm and a maximum Seebeck coefficient of ∼ −119 μV/K. A second sample 4-b2-c with similar thickness as 3-b1-c was Cu-contacted on both sides. As visible in Figure 8b, the resulting Seebeck coefficient is constant throughout TE, similar to the results shown in Figure 7, but lower with *S* ∼ −140 μV/K and thus *n* = 2.2 × 10^20^ cm^–3^. However, while the carrier concentrations before and after contacting are different between the sample groups, the change in carrier concentration upon contacting is comparable (Δ*n* = 1.4 × 10^20^ cm^–3^ for 3-b1-c sample in Figure 7a and Δ*n* = 1.7 × 10^20^ cm^–3^ for the 4-b2-c sample in Figure 8b).

**Figure 9:**
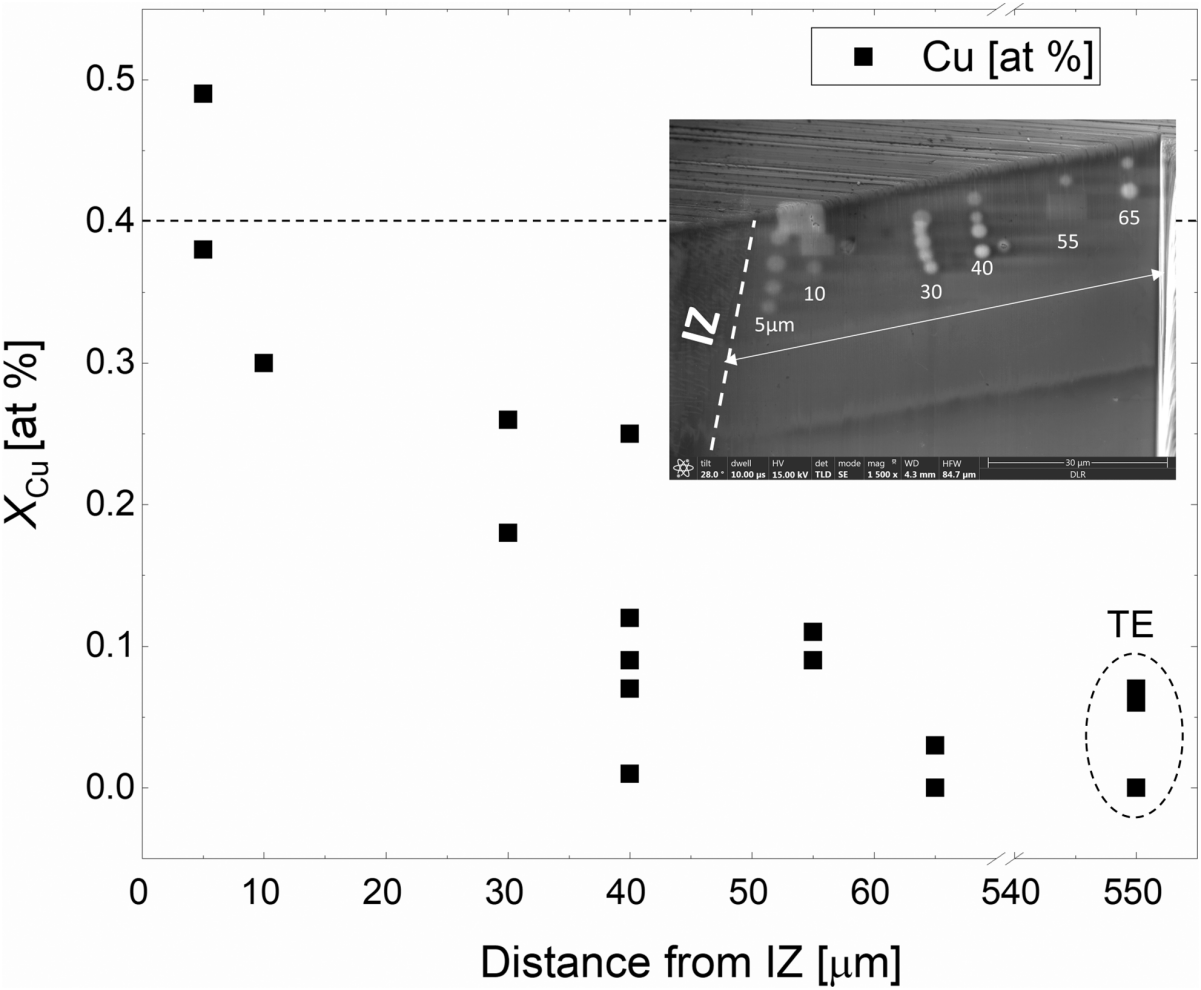
Calibrated Cu-WDS measurements on the sample 4-b2-c. The attached SEM image shows the measurement spots in the zoom-in 1, as presented in Figure 5. The dashed line indicates the amount of Cu required to explain the observed changes in the Seebeck coefficient by the formation of Cu-related point defects, pointing out that Cu diffusion into the sample cannot be the origin of the observed changes.

To further differentiate between Mg and Cu diffusion, the 4-b2-c sample underwent WDS point analysis, with measurements taken on the two FIB-milled regions shown in Figure 5. Figure 9 presents the WDS results showing the Cu concentration for positions ranging from 5 to 65 μm within zoom-in 1 from Figure 5. Multiple measurements were taken at each spot to obtain robust statistical data. Only measurements where elemental sums are within 100 wt.% ± 2 wt.% are included to ensure good data quality. This approach can reduce the number of data points for a given position; for example, the graph shows only one measurement spot for 10 μm, as the others were outside the 2% range. WDS shows deviation from the total 100 wt.% because, in this measurement, only Cu was calibrated for WDS, while all other elements were calibrated and measured by EDX. The non-homogeneity of the sample phases seen at such high magnification further contributed to this deviation. These measurement spots appear bright in the SEM image in Figure 9, likely due to electron beam exposure. This phenomenon is typically seen in Mg_2_(Si, Sn) samples but not in others, the reasons for which remain unclear. Multiple measurements were also taken inside the milled area of zoom-in 2 (Figure 5), i.e., far away from the interface.

As seen in Figure 9, all measurement points are plotted according to their sample positions, as indicated along the *x*-axis. The measurement results for the Cu concentration show some scatter and a clearly decreasing trend from 5 to 65 μm with respect to the boundary of the farthest Cu containing phase of the IZ, as shown in the zoom-in picture in Figure 9. Notably, at 65 μm, we measure <0.1 at.% Cu, which is also observed deep within the TE material (∼550 μm). The non-zero measurement result could be due to the WDS detection limit of <0.1 at.% or the distribution of a small amount of Cu within the sample.

Cu is detectable (>0.1 at.%) away from the interface only up to approximately 65 μm, which corresponds approximately to the first measured data point within the TE material of the representative Seebeck line scan in Figure 8b, as the step width is 100 μm. However, we observe a significant reduction in charge carrier concentration throughout the TE material, not only within the first 65 μm away from the interface.

To rule out that the measured changes in Seebeck coefficient are due to Cu amounts below the detection limit of WDS, we employ a simple defect chemistry model. Mg_2_(Si, Sn) has 12 atoms per unit cell; given the lattice constant of 0.66 nm, the atom density of the TE unit cell can be calculated as 12/(0.66 × 10^–9^ m)^3^ = 4.2 × 10^22^ cm^–3^. Considering the observed change in carrier concentration Δ*n* of 1.7 × 10^20^ cm^–3^, and assuming each Cu atom accepts one electron (most stable charged defect Cu_Mg_^1–^^29^), we would need 1.7 × 10^20^ Cu atoms per cm^3^ to explain the change in the observed change in *S* in Figure 8b, corresponding to *X*_Cu_ = 1.7 × 10^20^ cm^–3^/4.2 × 10^22^ cm^–3^ = 0.4 at.%.

Thus, if Cu diffusion into the TE was the cause of the observed change in *n*, we should measure approximately 0.4 at.% Cu. However, the WDS measurements (Figure 9), with a higher compositional resolution compared to EDS, reveal Cu concentrations well below this threshold, with a clear decreasing trend into depth of the material. Therefore, Cu diffusion into the TE can be ruled out as the origin of the observed change in carrier concentration in the inner of the TE sample. This suggests that the observed change in the *S* after contacting is primarily due to the loss of Mg diffusing into the Cu electrode, rather than Cu diffusing into the TE.

## 4 Discussion

This study employed Al_2_O_3_ ALD coating on non-contacted and Cu-contacted n-Mg_2_(Si, Sn) samples. Initial SEM characterization confirmed the coating’s uniformity and conformality. Subsequently, after subjecting all samples to a temperature of 723 K for a duration of 1 week within an Ar atmosphere, annealed sample surfaces were characterized by XRD and SEM. We observe the formation of MgO and traces of elemental Sn and Si content. In the literature,^38,44^ the mechanism of formation of MgO and evolution of decomposition was proposed based on aging studies conducted on uncoated samples. They have shown that Sn-rich Mg_2_(Si, Sn) phases decompose more rapidly and at lower temperatures than Si-rich phases. Additionally, the decomposition and oxidation tend to self-propagate from the surface to the interior of the sample. Zhang *et al.*(38) also reported that at 700 K, Mg in the material oxidizes due to residual O_2_ in the inert gas, driven by the melting of the Sn-rich phase, which accelerates Mg migration and oxidation. Both reported mechanisms are particularly relevant to our investigation of Sn-rich Mg_2_(Si, Sn) at high temperatures and to understand the formation of MgO as a result of annealing treatment. The novelty of our study lies in its focus on coated samples, which were annealed within an Ar atmosphere for 1 week. Zhang *et al.*(38) who studied the oxidation resistance of Al_2_O_3_-coated Mg_2_Si_0.4_Sn_0.6_ at 823 K for 12 h reported stable TE properties upon temperature cycling in Ar gas. However, they removed the surface layers before conducting TE measurements and did not report any microstructural investigation of the coating before and after annealing. In contrast, our present study conducts all TE measurements on samples with the coating intact and includes a detailed microstructural investigation of the coating both before and after annealing (Figures 3 and 4). We observed cracking of the alumina coating in areas where MgO formation is present. At this point, it remains unclear whether the cracking result from a CTE mismatch, which subsequently leads to MgO formation, or if the MgO formation itself causes the coating to crack. With respect to the cause of the cracking, Mounib *et al.*(45) deduced the reaction of Mg with Al_2_O_3_ at 823 K from DSC measurements. Given that n-type Mg_2_(Si, Sn) usually contains loosely bound Mg due to employing Mg excess,^30,41,46,47^ it is plausible that loosely bound Mg diffusing from within the grains of the TE material and then reacting with the Al_2_O_3_ plays a role in the formation of the observed cracks, besides the influence of the CTE mismatch between Al_2_O_3_ and the TE material. In agreement with the literature report, we thus confirm that the Al_2_O_3_ ALD coating enhances significantly the stability of non-contacted n-Mg_2_(Si, Sn), as the integral TE properties are only marginally affected, while uncoated samples have been shown to experience a drastic increase in the (absolute) Seebeck coefficient and a corresponding reduction in carrier concentration of up to 2 × 10^20^ cm^–3^when annealed at 710 K.^41^ Together with the recent finding of high diffusivity of Mg in Sn-rich solid solution compositions and consequent material degradation already at room temperature,^30,41^ this indicates that Mg loss to the environment (by oxidation or sublimation) is the most severe challenge to material stability. We show that ALD coating can be successfully used on TE materials and on functionalized legs to address these challenges, indicating a path not only toward stabilization of the TE material but also to stable devices.^48^ However, the observation of MgO coinciding with instances of cracked coating indicates the need for further research. Alternative approaches to protect TE materials and in particular Mg_2_*X* include SiO_2_ coatings,^49^ amorphous SiOC (black glass),^50^ solvent-based resins,^51^ and β-FeSi_2_,^52^ which have all been tested with Mg_2_Si and demonstrated improved material stability.

Regarding Cu-contacted samples, there is a keen interest to establish a methodology that differentiates between Mg loss and Mg-electrode interdiffusion at the interface. Therefore, Cu-contacted samples with and without ALD coating were assessed in comparison by estimating the loss of charge carrier concentration after contacting and after subsequent annealing. Local Seebeck coefficient measurements reveal a large decrease in carrier concentration after contacting and differential studies on a thin and a thick sample first confirm an electrode related interdiffusion process as main degradation mechanism by showing a charge carrier gradient for the thick sample and second indicate that the interdiffusion process is fast with 10 min at 873 K being sufficient to deplete a ∼ 1 mm thick sample. Further annealing of a thin contacted sample leads to only a minimal further change in Seebeck coefficient.

It was seen that the ALD coated, annealed Cu-contacted sample also experiences a minor change of Δ*n* ∼ 0.13 × 10^20^ cm^–3^ with respect to the Cu-contacted TE sample before coating and annealing, again showing that Mg loss has been strongly inhibited. Figure 6 and the corresponding Δ*n* values further confirm that the degradation observed in the annealed contacted sample is primarily attributed to either Cu diffusion and incorporation in the TE material and/or Mg diffusion out of the TE material, potentially into the IZ between Cu and TE material. Mg–Cu interdiffusion has been identified as the initial degradation mechanism during the contacting process, and annealing can further enhance Mg–Cu interdiffusion. This was further confirmed by WDS quantification measurement on a Cu-contacted sample, which shows a decreasing *X*_Cu_ trend moving away from the IZ into the TE material. Beyond 65 μm, Cu presence is negligible and significantly smaller than the level required to explain the observed change in carrier concentration, suggesting that Cu does not significantly influence the loss of carrier concentration. Since the *X*_Cu_ decreases only up to approximately 65 μm, corresponding to the first measured data point within the TE material of the representative Seebeck line scan in Figure 8b, its effect should be limited to this range. However, we observe a significant reduction in charge carrier concentration throughout the cross section of the TE material, which cannot be attributed solely to Cu diffusion. From simple defect chemistry calculation, we found that we require approximately 0.4 at.% of Cu to cause the observed change in *n*. We are well below 0.1 at.% of Cu (Figure 9) as we move from 65 μm to the middle of the TE. This indicates that the observed change in the Seebeck coefficient after contacting is primarily due to Mg out-diffusion into the Cu electrode rather than Cu diffusion into the TE. This work aligns with the findings of Ayachi *et al.*,^7^ who highlighted that Mg is the primary diffusing species into Cu, with localized Sn regions present, while Si is entirely absent. Previous studies on Cu–Mg interdiffusion at around 723 K, such as those by Dai *et al.*,^53^ indicate the formation of Mg_2_Cu and MgCu_2_ binary phases, with Mg_2_Cu forming first due to its lower activation energy. Their research also reports activation energies for Cu in Mg and Mg in Cu as 164.04 ± 7.18 and 139.38 ± 0.65 kJ/mol, respectively. While these numbers confirm that Mg diffusion is the dominant mechanism in the interdiffusion zone, we study the changes in the TE material (not IZ) and show that it is Mg out-diffusion from the TE material rather than Cu diffusion into it, that drives these changes and eventually impacts the intrinsic TE properties beyond the visible interdiffusion layers.

A further confirmation for Mg out-diffusion rather than Cu diffusion into TE is that the two Cu-contacted samples with the same thicknesses reported in Figures 7 and 8 have different starting and resulting Seebeck coefficients (−178 μV/K from batch 1 vs −140 μV/K to batch 2 after contacting) but a comparable change in carrier concentration Δ*n* = 1.4 × 10^20^ cm^–3^ for batch 1 and Δ*n* = 1.7 × 10^20^ cm^–3^ for the sample from batch 2. If we assume that the change in carrier concentration was due to Cu diffusing into the TE material, the resulting charge carrier concentration would be given approximately by the crossing point between Cu and Bi defect formation energies,^29^ provided there is an abundant supply of Cu. One would thus expect both samples to have the same carrier concentration after equilibration, which is not observed. However, if we assume Mg diffusing into Cu (or the IZ), the expected change in carrier concentration is given by the difference between lower and upper solubility limit of Mg in the TE material, so Δ*n* (but not the final *n*) should be the same for all samples, which is close to the experimental observation.

## 5 Conclusions

We have investigated the degradation mechanisms during the operation of n-type Mg_2.06_Si_0.3_Sn_0.665_Bi_0.035_ material samples, as well as its functionalized legs with Cu as an electrode under high temperature operating conditions. In agreement with earlier work, we find that the samples show a drastic deterioration of their thermoelectric performance after contacting and after annealing. Our results show that ALD coating by Al_2_O_3_ increases the material stability of non-contacted samples enormously, presumably due to suppressing Mg loss from the sample by sublimation or oxidation, which leads to a change in intrinsic defect concentrations and hence to performance degradation. Analysis of local and integral TE properties of functionalized samples reveals a charge carrier loss (preferentially at the interface) even if Mg loss by sublimation is suppressed. With WDS measurements, we further dive deeper into the mechanism of Mg–Cu interdiffusion and show that we have negligible Cu diffusion into the TE material, indicating Mg loss into the electrode as the main degradation mechanism for Cu-contacted samples. While electrode-induced defects in TE materials have been previously identified, this study is the first to experimentally demonstrate the relevance of the opposite mechanism. By combining ALD coating to inhibit Mg loss with microstructural analysis, we present a novel methodology to investigate such phenomena. Our findings highlight that focusing on developing a diffusion barrier for Mg, separating Cu from the TE material, could pave the way for achieving stable and high-performance Mg_2_*X*-based TEGs.

## Supporting Information Available

XRD on bulk TE sample (0-b1) with Co-source (Figure S1); initial examination of ALD-coated samples under a light microscope (Figure S2); and mass % of an EDS point analysis of Figure 3 (Table S1) (PDF)
