# Supplementary material for: On the Origin of Temperature Induced Performance Degradation of Cu-Contacted Mg2X-Based (X = Si, Sn) Thermoelectric Materials
Source: ACS Appl Mater Interfaces. 2025 Apr 29;17(19):28777–88. doi: 10.1021/acsami.5c00258 (PMC12086763; doi:10.1021/acsami.5c00258)
Supplement: Supplementary file 1 — am5c00258_si_001.pdf [file am5c00258_si_001.pdf]

Supporting Information

On the Origin of Temperature Induced Performance Degradation of Cu-Contacted Mg_2X-Based (X= Si, Sn) Thermoelectric Materials

Radhika Deshpande[1*], Amin Bahrami[2], Frederic Kreps[1], Ran He[2], Pingjun Ying[2], Kornelius Nielsch[2,3], Eckhard Müller[1,4], Johannes de Boor[1,5]

1. Institute of Materials Research, German Aerospace Centre (DLR), 51147 Cologne, Germany
2. Leibniz Institute of Solid State and Materials Science, 01069 Dresden, Germany
3. Institute of Materials Science, Technische Universität Dresden, 01062 Dresden, Germany
4. Institute of Inorganic and Analytical Chemistry, Justus Liebig University of Giessen, 35392 Giessen, Germany
5. Institute of Technology for Nanostructures (NST) and CENIDE, Faculty of Engineering, University of Duisburg-Essen, 47057 Duisburg, Germany

Corresponding author: radhika.deshpande@dlr.de

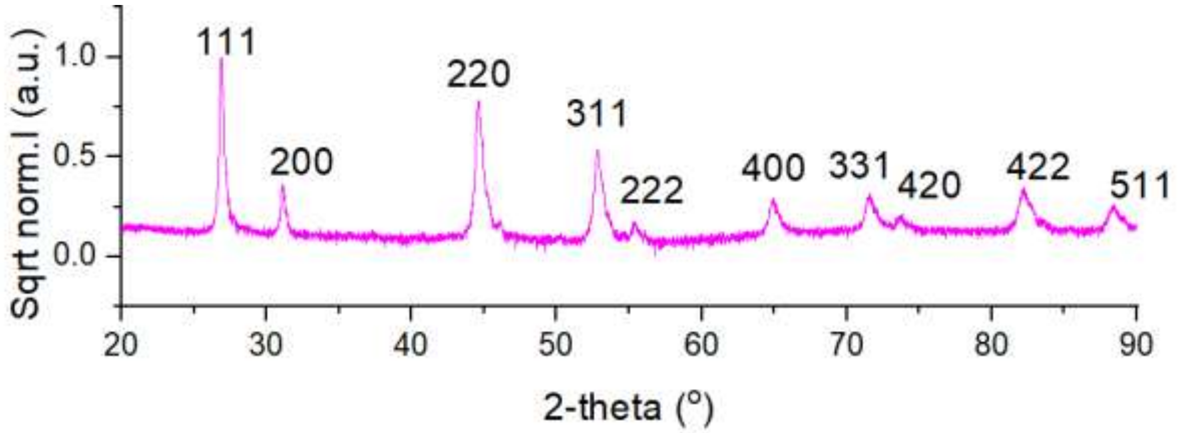

Figure S1: XRD on bulk TE sample (0-b1) with Co-source, taken with step size of 0.01° and time per step of 1 s. All peaks of Mg_2X sample are identified within cubic Fm-3m space group. No reflections corresponding to MgO were observed at approximately 50.4° and 74.2°. These peaks were predicted based on Bragg's law calculations using the standard MgO peak pattern for the Cu-Kα source, where the primary peaks are at ~ 42.9° and ~ 62.5°, respectively. This confirms that the initial bulk sample, 0-b1 is phase-pure with no MgO impurities.

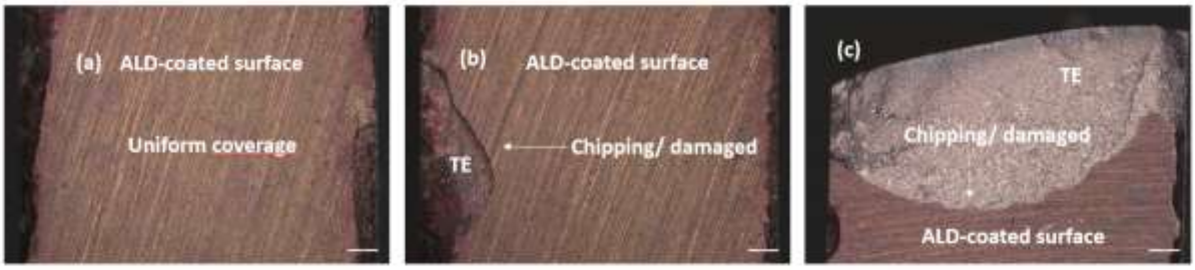

Figure S2: Initial examination of ALD-coated samples under light microscope with (a) showing uniform coating coverage and (b-c) showing a contrast in color at the TE

material-coating layer interface where the coating has chipped off, further confirming the uniform coating of the selected samples.

Table S1: Mass% of an EDS point analysis of Figure 3

**Element**	**Mass %**
O	40.63
Mg	9.41
Al	36.44
Si	1.44
Sn	11.45
Bi	0.63
Total	100
